# Multiplicity and asymptotic behavior of solutions for Kirchhoff type equations involving the Hardy–Sobolev exponent and singular nonlinearity

**DOI:** 10.1186/s13660-018-1806-8

**Published:** 2018-08-16

**Authors:** Liejun Shen

**Affiliations:** School of Mathematics and Statistics, Central China Normal University, Wuhan, China

**Keywords:** 35J47, 35J60, Hardy–Sobolev exponent, Kirchhoff, Multiplicity, Nehari manifold, Fibering map, Asymptotic behavior, Mountain pass theorem, Ekeland’s variational principle

## Abstract

In this paper, we study a class of critical elliptic problems of Kirchhoff type:

$$ \biggl[a+b \biggl( \int_{\mathbb{R}^{3}}\vert \nabla u\vert ^{2}-\mu \frac{u^{2}}{\vert x\vert ^{2}}\,dx \biggr)^{\frac{2-\alpha }{2}} \biggr]\biggl(-\Delta u- \mu \frac{u}{\vert x\vert ^{2}}\biggr) = \frac{\vert u\vert ^{2^{*}(\alpha )-2}u }{\vert x\vert ^{\alpha }}+\lambda \frac{f(x)\vert u\vert ^{q-2}u }{\vert x\vert ^{\beta }}, $$

where $a,b>0$, $\mu \in [0,1/4)$, $\alpha , \beta \in [0,2)$, and $q\in (1,2)$ are constants and $2^{*}(\alpha )=6-2\alpha $ is the Hardy–Sobolev exponent in $\mathbb{R}^{3}$. For a suitable function $f(x)$, we establish the existence of multiple solutions by using the Nehari manifold and fibering maps. Moreover, we regard $b>0$ as a parameter to obtain the convergence property of solutions for the given problem as $b\searrow 0^{+}$ by the mountain pass theorem and Ekeland’s variational principle.

## Introduction and main results

In the present paper, we consider the following Schrödinger equation:

1.1 $$\begin{aligned}& \biggl[a+b \biggl( \int_{\mathbb{R}^{3}}\vert \nabla u\vert ^{2}-\mu \frac{u^{2}}{\vert x\vert ^{2}}\,dx \biggr)^{\frac{2-\alpha }{2}} \biggr]\biggl(-\Delta u- \mu \frac{u}{\vert x\vert ^{2}}\biggr) \\& \quad = \frac{\vert u\vert ^{2^{*}(\alpha )-2}u }{\vert x\vert ^{\alpha }}+\lambda \frac{f(x)\vert u\vert ^{q-2}u }{\vert x\vert ^{\beta }}, \end{aligned}$$

where $a,b>0$, $\mu \in [0,1/4)$, $\alpha ,\beta \in [0,2)$, and $q\in (1,2)$ are constants and $2^{*}(\alpha )=6-2\alpha $ is the critical Hardy–Sobolev exponent.

We call (1.1) a Schrödinger equation of Kirchhoff type because of the appearance of the term $b(\int_{\mathbb{R}^{3}}|\nabla u|^{2}-\mu u^{2}|x|^{-2}\,dx)^{{{(2-\alpha )}/{2}}}$ which makes the study of (1.1) interesting. Indeed, if we choose $\mu = \alpha =0$ and let $|u|^{4}u+f(x)|u|^{q-2}u|x|^{-\beta }=k(x,u)-V(x)u$, then (1.1) transforms to the following classical Kirchhoff type equation:

1.2 $$ - \biggl(a+ b \int_{\mathbb{R}^{3}}\vert \nabla u\vert ^{2}\,dx \biggr) \Delta u+V(x)u=k(x,u) $$

which is degenerate if $b=0$ and non-degenerate otherwise. Equation (1.2) arises in a meaningful physical context. In fact, if we set $V(x)=0$ and replace $\mathbb{R}^{3}$ by a bounded domain $\Omega \subset \mathbb{R}^{3}$, then we get the following Dirichlet problem:

$$ - \biggl(a+ b \int_{\Omega }\vert \nabla u\vert ^{2}\,dx \biggr) \Delta u=k(x,u) $$

which is related to the stationary analogue of the equation

$$ \rho \frac{\partial^{2}u}{\partial t^{2}}- \biggl(\frac{P_{0}}{h}- \frac{E}{2L} \int_{0}^{L} \biggl\vert \frac{\partial u}{\partial x} \biggr\vert ^{2}\,dx \biggr)\frac{\partial^{2}u}{\partial x^{2}}=0 $$

proposed by Kirchhoff in [16] as an extension of the classical D’Alembert’s wave equation for free vibrations of elastic strings. This model takes the changes in length of the string produced by transverse vibrations into account. After J. L. Lions in his pioneer work [21] presented an abstract functional analysis framework to (1.2), this problem has been widely studied in extensive literature such as [8, 11, 12, 19, 20, 24, 25].

In their celebrated paper, Ambrosetti et al. [2] studied the following semilinear elliptic equation with concave-convex nonlinearities:

$$ \textstyle\begin{cases} - \Delta u = \vert u\vert ^{p-2}u+\xi \vert u\vert ^{q-2}u, &\text{in } \Omega , \\ u=0, & \text{on } \partial \Omega , \end{cases} $$

where Ω is a bounded domain in $\mathbb{R}^{N}$, $\xi >0$ and $1< q<2<p\leq 2^{*}={2N}/{(N-2)}$ with $N\geq 3$. By the variational method, they obtained the existence and multiplicity of positive solutions to the above problem. Subsequently, an increasing number of researchers have paid attention to semilinear elliptic equations with critical exponent and concave-convex nonlinearities; for example, see [1, 5, 13, 14, 27, 29] and the references therein.

Using the Nehari manifold and fibering maps, Chen et al. [6] extended the above analysis to the subcritical semilinear elliptic problem of Kirchhoff type:

$$ \textstyle\begin{cases} - M (\int_{\Omega }\vert \nabla u\vert ^{2}\,dx )\Delta u =g(x)\vert u\vert ^{p-2}u+ \lambda h(x)\vert u\vert ^{q-2}u\quad {\text{in }}{\Omega }, \\ u = 0\quad {\text{on }}\partial \Omega , \end{cases} $$

where *M* is the so-called Kirchhoff function depending on $1< q<2<p<2^{*}$, Ω is a bounded domain with a smooth boundary in $\mathbb{R}^{N}$ and the weight functions $h,g\in C(\overline{ \Omega })$ satisfy some specified conditions

$$ f^{\pm }=\max \{\pm f,0\}\neq 0\quad \text{and}\quad g^{\pm }= \max \{ \pm g,0\}\neq 0, $$

they proved the existence of multiple solutions of it. In the critical case, Lei et al. [19] considered the following Kirchhoff problem in three dimensions:

$$ \textstyle\begin{cases} - (a+\epsilon \int_{\Omega }\vert \nabla u\vert ^{2}\,dx )\Delta u = u ^{5}+\lambda u^{q-1}\quad {\text{in }}{\Omega }, \\ u = 0\quad {\text{on }}\partial \Omega , \end{cases} $$

where $\epsilon >0$ is a sufficiently small constant, and they employed the mountain pass theorem to show that the problem admits at least two different positive solutions. Some other related and important results can be found in [18, 23] and the references therein.

Before stating our main results, we introduce some function spaces. Throughout the paper, $L^{p}(\mathbb{R}^{3})$ ($1\leq p\leq +\infty $) is the usual Lebesgue space with the standard norm $|u|_{p}$, and we consider the Hilbert space $D^{1,2}(\mathbb{R}^{3})$ equipped with its usual inner product and norm

$$ (u,v)_{D^{1,2}(\mathbb{R}^{3})}= \int_{\mathbb{R}^{3}}\nabla u\nabla v\,dx \quad \text{and}\quad \Vert u \Vert _{D^{1,2}(\mathbb{R}^{3})}= \biggl( \int_{\mathbb{R}^{3}}\vert \nabla u\vert ^{2}\,dx \biggr)^{\frac{1}{2}}. $$

By the well-known Hardy inequality [17]

$$ \int_{\mathbb{R}^{3}}\frac{u^{2}}{\vert x\vert ^{2}}\,dx\leq 4 \int_{\mathbb{R} ^{3}}\vert \nabla u\vert ^{2}\,dx, $$

we derive that the induced inner product and norm

$$ (u,v)= \int_{\mathbb{R}^{3}}\nabla u\nabla v-\mu \frac{uv}{\vert x\vert ^{2}}\,dx \quad \text{and}\quad \Vert u\Vert = \biggl( \int_{\mathbb{R}^{3}}\vert \nabla u\vert ^{2}- \mu \frac{u^{2}}{\vert x\vert ^{2}}\,dx \biggr)^{\frac{1}{2}} $$

are equivalent to the usual inner product and norm on $D^{1,2}( \mathbb{R}^{3})$ for any $\mu \in [0,1/4)$. As a special case of [15, Lemma 2.3], for any $\mu \in [0,1/4)$ and $s\in [0,2)$, we can define

1.3 $$ S_{\mu ,s}= \biggl\{ \int_{\mathbb{R}^{3}}\vert \nabla u\vert ^{2}-\mu \frac{u ^{2}}{\vert x\vert ^{2}}\,dx:u\in D^{1,2}\bigl(\mathbb{R}^{3}\bigr) \text{ and } \int_{\mathbb{R}^{3}}\frac{\vert u\vert ^{2^{*}(s)}}{\vert x\vert ^{s}}\,dx=1 \biggr\} . $$

We also know that $S_{\mu ,s}$ can be attained by a positive function $U\in D^{1,2}(\mathbb{R}^{3})$ satisfying

1.4 $$ \int_{\mathbb{R}^{3}}\vert \nabla U\vert ^{2}-\mu \frac{U^{2}}{\vert x\vert ^{2}}\,dx= \int_{\mathbb{R}^{3}}\frac{\vert U\vert ^{2^{*}(s)}}{\vert x\vert ^{s}}\,dx=S_{\mu ,s}^{ \frac{3-s}{2-s}}. $$

Motivated by all the works mentioned above, we are interested in the multiplicity and asymptotic behavior of solutions of (1.1) whose natural variational functional is

$$ J(u)=\frac{a}{2}\Vert u\Vert ^{2}+\frac{b}{4-\alpha }\Vert u\Vert ^{4-\alpha } -\frac{1}{2^{*}( \alpha )} \int_{\mathbb{R}^{3}}\vert u\vert ^{2^{*}(\alpha )}\vert x\vert ^{-\alpha }\,dx-\frac{ \lambda }{q} \int_{\mathbb{R}^{3}}f(x)\vert u\vert ^{q}\vert x\vert ^{-\beta }\,dx. $$

Note that we can adopt the idea used in [28] to prove that $J(u)$ is well-defined on $D^{1,2}(\mathbb{R}^{3})$ and of class $C^{1}$. Furthermore, any solution of (1.1) is a critical point of $J(u)$. Hence we obtain the solutions of it by finding the critical points of the functional $J(u)$. To this aim, we assume the following condition:

(F) $0\lvertneqq f(x)\in L^{\infty }(\mathbb{R}^{3})$ and there exists $R_{0}>0$ such that $\operatorname{supp}f\in B_{R_{0}}(0)$.

Since $\operatorname{supp}f\subset B_{R_{0}}(0)$, using Hölder’s inequality and (1.3), we have

1.5 $$\begin{aligned} \int_{\mathbb{R}^{3}}\frac{f(x)\vert u\vert ^{q}}{\vert x\vert ^{\beta }}\,dx \leq& \vert f\vert _{ \infty } \biggl( \int_{B_{R_{0}}(0)}\frac{1}{\vert x\vert ^{\beta }}\,dx \biggr)^{\frac{2^{*}( \beta )-q}{2^{*}(\beta )}} \biggl( \int_{B_{R_{0}}(0)}\frac{\vert u\vert ^{2^{*}( \beta )}}{\vert x\vert ^{\beta }}\,dx \biggr)^{\frac{q}{2^{*}(\beta )}} \\ \triangleq& \vert f\vert _{\infty }C_{R_{0},\beta ,q} \biggl( \int_{B_{R_{0}}(0)}\frac{\vert u\vert ^{2^{*}( \beta )}}{\vert x\vert ^{\beta }}\,dx \biggr)^{\frac{q}{2^{*}(\beta )}} \\ \leq& \vert f\vert _{ \infty }C_{R_{0},\beta ,q} S_{\mu ,\beta }^{-\frac{q}{2}} \Vert u\Vert ^{q}. \end{aligned}$$

For the convenience of narration, we set

$$\begin{aligned}& \lambda_{1}\triangleq \frac{2(2-\alpha )\sqrt{2ab} S_{\mu ,\beta } ^{\frac{q}{2}}}{ (2^{*}(\alpha )-q )\vert f^{+}\vert _{\infty }C_{R_{0}, \beta ,q}} \biggl[\frac{2\sqrt{ab(2-q)(4-\alpha -q)}S_{\mu ,\alpha } ^{\frac{2^{*}(\alpha )}{2}}}{2^{*}(\alpha )-q} \biggr]^{\frac{6-\alpha -2q}{6-3\alpha }}>0, \\& \lambda_{2}\triangleq \frac{a(4-2\alpha )S_{\mu ,\beta }^{\frac{q}{2}}}{ (2^{*}(\alpha )-q )\vert f^{+}\vert _{\infty }C_{R_{0},\beta ,q}} \biggl[\frac{a(2-q)S _{\mu ,\alpha }^{\frac{2^{*}(\alpha )}{2}}}{2^{*}(\alpha )-q} \biggr]^{\frac{2-q}{4-2 \alpha }}>0, \\& \lambda_{3}\triangleq \frac{1}{2}(aS_{\mu ,\alpha })^{\frac{(3-\alpha )(2-q)}{2(2-\alpha )}} \biggl[\frac{2-\alpha }{2C_{0}(3-\alpha )} \biggr]^{\frac{2-q}{2}}>0, \\& \lambda_{4}\triangleq \biggl(\frac{t_{1}^{q}\int_{\mathbb{R}^{3}}f(x)\vert U\vert ^{q}\vert x\vert ^{- \beta }\,dx}{C_{0}q} \biggr)^{\frac{2-q}{q}}>0, \\& \lambda_{5}=\frac{aqS_{\mu ,\beta }^{\frac{q}{2}}(2^{*}(\alpha )-2)}{2\vert f\vert _{ \infty }C_{R_{0},\beta ,q}(2^{*}(\alpha )-q)} \biggl[\frac{a2^{*}( \alpha )S_{\mu ,\alpha }^{\frac{2^{*}(\alpha )}{2}}(2-q)}{2(2^{*}( \alpha )-q)} \biggr]^{\frac{2-q}{2^{*}(\alpha )-2}}>0, \\& \Lambda_{1}\triangleq \max \{\lambda_{1}. \lambda_{2}\}, \\& \Lambda _{2}\triangleq \max \bigl\{ {q \lambda_{1}}/{\sqrt{2(4-\alpha )}},{q\lambda _{2}}/{2}\bigr\} , \end{aligned}$$

and

$$ \Lambda_{*}\triangleq \min \{\Lambda_{1}, \lambda_{3},\lambda_{4}\},\qquad \Lambda_{**} \triangleq \min \{\Lambda_{2},\lambda_{3}, \lambda_{4}\},\qquad \Lambda_{M}\triangleq \min \{ \lambda_{3},\lambda_{4},\lambda_{5} \}, $$

where $C_{0}>0$ is given by Lemma 3.3 and $t_{1}\in (0,1)$ only depends on $\lambda_{3}$.

### Remark 1.1

It is easy to see that the constants $\lambda_{i}$ for $i\in \{3,4,5 \}$ are independent of *b*, and then $\Lambda_{M}$ is also independent of *b*.

We are ready to state our first result.

### Theorem 1.2

*Assume* (F), $\mu \in [0,1/4)$, $\alpha ,\beta \in [0,2)$, *and* $q\in (1,2)$, *then for any* $a,b>0$ *problem* (1.1) *admits at least one positive solution for* $\lambda \in (0,\Lambda_{*})$ *and two positive solutions for* $\lambda \in (0,\Lambda_{**})$.

### Remark 1.3

If the whole space $\mathbb{R}^{3}$ is replaced by a bounded domain Ω and $f(x)\equiv 1$ with $\beta =0$, Theorem 1.2 can be seen as an improvement of the main results in [3, 7]. On the other hand, Theorem 1.2 extends the results of [6] to a more general case.

Inspired by the works in [8, 24, 25], we prefer to study the asymptotic behavior of multiple solutions to (1.1) because the solutions depend on the parameter *b*. By analyzing the convergence property, we establish the following result in this paper.

### Theorem 1.4

*Assume* (F), $\mu \in [0,1/4)$, $\alpha ,\beta \in [0,2)$, *and* $q\in (1,2)$, *then* (1.1) *has at least two positive solutions* $u_{b}^{1}$ *and* $u_{b}^{2}$ *for any* $\lambda \in (0,\lambda _{M})$. *Moreover*, *let* $\lambda \in (0,\lambda_{M})$ *and* $a>0$ *be fixed constants*, *then there exist subsequences still denoted by themselves* $\{u_{b}^{1}\}$ *and* $\{u_{b}^{2}\}$ *such that* $u_{b}^{i}\to u^{i}$ *in* $D^{1,2}(\mathbb{R}^{3})$ *as* $b\searrow 0^{+}$ *for* $i\in \{1,2\}$, *where* $u^{1}$ *and* $u^{2}$ *are two nontrivial solutions of*

1.6 $$ a \biggl(-\Delta u-\mu \frac{u}{\vert x\vert ^{2}} \biggr) = \frac{\vert u\vert ^{2^{*}(\alpha )-2}u }{\vert x\vert ^{\alpha }}+\lambda \frac{f(x)\vert u\vert ^{q-2}u }{\vert x\vert ^{\beta }}. $$

### Remark 1.5

A natural question is why we do not study the convergence of solutions obtained in Theorem 1.2. In fact, if we do this step by step, we can only prove that equation (1.6) has at least one nontrivial solution. The main reason for this phenomenon is that we cannot prove there exists $d_{1}<0$ independent of *b* such that $m^{+}< d_{1}$ (see Lemma 2.5 for details). To explain this in a little more detail, we assume there exists a sequence $\{u_{b}\} \subset D^{1,2}(\mathbb{R}^{3})$ of solutions of (1.1) satisfying $J(u_{b})=m^{-}<0$. By a standard method, we can show that there exists $u\in D^{1,2}(\mathbb{R}^{3})$ such that $u_{b}\to u$ in $D^{1,2}(\mathbb{R}^{3})$ as $b\to 0^{+}$. Unfortunately, we fail to prove $m^{+}\nrightarrow 0$ as $b\to 0^{+}$, which yields $u\neq 0$.

The outline of this paper is as follows. In Sect. 2, we present some preliminary results. In Sect. 3, we obtain the existence of two local minimax solutions of (1.1). In Sect. 4, we prove the convergence property on the parameter $b>0$.

### Notations

Throughout this paper we shall denote by *C* and $C_{i}$ ($i=1, 2,\ldots $) various positive constants whose exact value may change from lines to lines but are not essential to the analysis of problem. We use “→” and “⇀” to denote the strong and weak convergence in the related function space, respectively. For any $\rho >0$ and any $x\in \mathbb{R}^{3}$, $B_{\rho }(x)$ denotes the ball of radius *ρ* centered at *x*, that is, $B_{\rho }(x):=\{y \in \mathbb{R}^{3}:|y-x|<\rho \}$.

Let $(X,\|\cdot \|)$ be a Banach space with its dual space $(X^{*},\| \cdot \|_{*})$, and Ψ be its functional on *X*. The Palais–Smale sequence at level $d\in \mathbb{R}$ ($(PS)_{d}$ sequence in short) corresponding to Ψ satisfies that $\Psi (x_{n})\to d$ and $\Psi^{\prime }(x_{n})\to 0$ as $n\to \infty $, where $\{x_{n}\} \subset X$.

## Nehari manifold and fibering map

In this section, we study the so-called Nehari manifold because the variational functional $J(u)$ is not bounded from below on $D^{1,2}( \mathbb{R}^{3})$. Let us define

$$ \mathcal{N}= \bigl\{ u\in D^{1,2}\bigl(\mathbb{R}^{3}\bigr) \backslash \{0\}: \bigl\langle J^{\prime }(u),u\bigr\rangle =0 \bigr\} , $$

and then any nontrivial solution of (1.1) belongs to $\mathcal{N}$. Obviously, $u\in \mathcal{N}$ if and only if

$$ a\Vert u\Vert ^{2}+b\Vert u\Vert ^{4-\alpha }- \int_{\mathbb{R}^{3}}\vert u\vert ^{2^{*}(\alpha )}\vert x\vert ^{-\alpha }\,dx- \lambda \int_{\mathbb{R}^{3}}f(x)\vert u\vert ^{q}\vert x\vert ^{- \beta }\,dx=0 \quad \text{and}\quad u\neq 0. $$

The following lemma tells us the behavior of $J(u)$ on $\mathcal{N}$.

### Lemma 2.1

*The functional* $J(u)$ *is coercive and bounded from below on* $\mathcal{N}$.

### Proof

For any $u\in \mathcal{N}$, since $\alpha \in (0,2)$ and $q\in (1,2)$, we get

$$ J(u)=J(u)-\frac{1}{2^{*}(\alpha )} \bigl\langle J^{\prime }(u),u\bigr\rangle \geq \frac{2-\alpha }{6-2\alpha }\Vert u\Vert ^{2} - \biggl(\frac{1}{q}- \frac{1}{2^{*}( \alpha )} \biggr)\vert f\vert _{\infty }C_{R_{0},\beta ,q} S_{\mu ,\beta }^{- \frac{q}{2}}\Vert u\Vert ^{q}, $$

which yields that $J(u)$ is coercive and bounded from below on $\mathcal{N}$. □

The Nehari manifold $\mathcal{N}$ is closely linked to the functions $\varphi_{u}(t)=J(tu)$ for any $t>0$. As we all know, the above maps were introduced by Drábek and Pohozaev [9] and discussed in Brown and Zhang [4] (or Chen et al. [6]). For any $u\in D^{1,2}(\mathbb{R}^{3})$, we have

$$\begin{aligned}& \varphi_{u}(t)=\frac{a}{2}t^{2}\Vert u\Vert ^{2}+\frac{b}{4-\alpha }t^{4- \alpha }\Vert u\Vert ^{4-\alpha } -\frac{t^{2^{*}(\alpha )}}{2^{*}(\alpha )} \int_{\mathbb{R}^{3}}\frac{\vert u\vert ^{2^{*}(\alpha )}}{\vert x\vert ^{\alpha }}\,dx -\frac{t ^{q}}{q}\lambda \int_{\mathbb{R}^{3}}\frac{f(x)\vert u\vert ^{q}}{\vert x\vert ^{\beta }}\,dx, \\& \varphi_{u}^{\prime }(t)=at\Vert u\Vert ^{2}+bt^{3-\alpha }\Vert u\Vert ^{4-\alpha } - t^{2^{*}(\alpha )-1} \int_{\mathbb{R}^{3}}\frac{\vert u\vert ^{2^{*}(\alpha )}}{\vert x\vert ^{ \alpha }}\,dx - t^{q-1}\lambda \int_{\mathbb{R}^{3}}\frac{f(x)\vert u\vert ^{q}}{\vert x\vert ^{ \beta }}\,dx, \\& \varphi_{u}^{\prime \prime }(t)=a\Vert u \Vert ^{2}+b(3-\alpha )t^{2-\alpha } \Vert u\Vert ^{4-\alpha } - \bigl(2^{*}(\alpha )-1 \bigr)t^{2^{*}(\alpha )-2} \int_{\mathbb{R}^{3}}\frac{\vert u\vert ^{2^{*}(\alpha )}}{\vert x\vert ^{\alpha }}\,dx \\& \hphantom{\varphi_{u}^{\prime \prime }(t)=}{}- (q-1)t^{q-2}\lambda \int_{\mathbb{R}^{3}}\frac{f(x)\vert u\vert ^{q}}{\vert x\vert ^{ \beta }} \,dx. \end{aligned}$$

It is easy to see that for any $u\in D^{1,2}(\mathbb{R}^{3})\backslash \{0\}$ and $t>0$ we obtain

$$ t\varphi_{u}^{\prime }(t)=at^{2}\Vert u\Vert ^{2}+bt^{4-\alpha }\Vert u\Vert ^{4- \alpha } - t^{2^{*}(\alpha )} \int_{\mathbb{R}^{3}}\frac{\vert u\vert ^{2^{*}( \alpha )}}{\vert x\vert ^{\alpha }}\,dx - t^{q}\lambda \int_{\mathbb{R}^{3}}\frac{f(x)\vert u\vert ^{q}}{\vert x\vert ^{ \beta }}\,dx, $$

which gives that $\varphi_{u}^{\prime }(t)=0$ if and only if $tu\in \mathcal{N}$. In particular, $\varphi_{u}^{\prime }(1)=0$ if and only if $u\in \mathcal{N}$. Arguing as Brown and Zhang [4], we split $\mathcal{N}$ into three parts:

$$\begin{aligned}& \mathcal{N}^{+}=\bigl\{ u\in \mathcal{N}: \varphi_{u}^{\prime \prime }(1)>0 \bigr\} , \\& \mathcal{N}^{0}=\bigl\{ u\in \mathcal{N}:\varphi_{u}^{\prime \prime }(1)=0 \bigr\} , \\& \mathcal{N}^{-}=\bigl\{ u\in \mathcal{N}:\varphi_{u}^{\prime \prime }(1)< 0 \bigr\} . \end{aligned}$$

Therefore, for any $u\in \mathcal{N}$, we have

2.1 $$\begin{aligned} \varphi_{u}^{\prime \prime }(1) &=a\Vert u\Vert ^{2}+b(3-\alpha )\Vert u\Vert ^{4- \alpha }-(5-2\alpha ) \int_{\mathbb{R}^{3}}\frac{\vert u\vert ^{2^{*}(\alpha )}}{\vert x\vert ^{ \alpha }}\,dx-(q-1)\lambda \int_{\mathbb{R}^{3}}\frac{f(x)\vert u\vert ^{q}}{\vert x\vert ^{ \beta }}\,dx \\ &=a(2-q)\Vert u\Vert ^{2}+b(4-\alpha -q)\Vert u \Vert ^{4-\alpha }- \bigl(2^{*}(\alpha )-q \bigr) \int_{\mathbb{R}^{3}}\frac{\vert u\vert ^{2^{*}(\alpha )}}{\vert x\vert ^{\alpha }}\,dx \end{aligned}$$

2.2 $$\begin{aligned} &=a(2\alpha -4)\Vert u\Vert ^{2}+b(\alpha -2) \Vert u\Vert ^{4-\alpha }+ \bigl(2^{*}( \alpha )-q \bigr)\lambda \int_{\mathbb{R}^{3}}\frac{f(x)\vert u\vert ^{q}}{\vert x\vert ^{ \beta }}\,dx. \end{aligned}$$

It is similar to the argument in Brown and Zhang [4, Theorem 2.3] that we can derive the following result.

### Lemma 2.2

*Suppose* $u\in D^{1,2}(\mathbb{R}^{3})$ *is a local minimizer for* $J(u)$ *on* $\mathcal{N}$ *and* $u\notin \mathcal{N}^{0}$, *then* $J^{\prime }(u)=0$ *in* $(D^{1,2}(\mathbb{R}^{3}))^{*}$.

Inspired by the above lemma, we will study when $\mathcal{N}^{0}= \emptyset $ is established.

### Lemma 2.3

*If* $0<\lambda <\Lambda_{1}\triangleq \max \{\lambda_{1},\lambda_{2}\}$, *then* $\mathcal{N}^{0}=\emptyset $.

### Proof

We argue it indirectly and assume that, for any $u\in \mathcal{N}^{0}$, using (2.1) and (2.2) we have

$$\begin{aligned}& 2\sqrt{ab(2-q) (4-\alpha -q)}\Vert u\Vert ^{\frac{6-\alpha }{2}} \\& \quad \leq a(2-q) \Vert u\Vert ^{2}+b(4-\alpha -q)\Vert u\Vert ^{4-\alpha } \\& \quad = \bigl(2^{*}(\alpha )-q \bigr) \int_{\mathbb{R}^{3}}\frac{\vert u\vert ^{2^{*}( \alpha )}}{\vert x\vert ^{\alpha }}\,dx \leq \bigl(2^{*}( \alpha )-q \bigr)S_{\mu , \alpha }^{-\frac{2^{*}(\alpha )}{2}}\Vert u\Vert ^{2^{*}(\alpha )} \end{aligned}$$

and by (1.5)

$$\begin{aligned} 2(2-\alpha )\sqrt{2ab}\Vert u\Vert ^{\frac{6-\alpha }{2}} \leq& a(4-2\alpha )\Vert u\Vert ^{2}+b(2-\alpha )\Vert u\Vert ^{4-\alpha } \\ =& \bigl(2^{*}(\alpha )-q \bigr)\lambda \int_{\mathbb{R}^{3}}\frac{f(x)\vert u\vert ^{q}}{\vert x\vert ^{ \beta }} \\ \leq& \bigl(2^{*}(\alpha )-q \bigr)\lambda \vert f\vert _{\infty }C_{R_{0}, \beta ,q}S_{\mu ,\beta }^{-\frac{q}{2}}\Vert u\Vert ^{q}, \end{aligned}$$

which yields that

2.3 $$ \biggl[\frac{2\sqrt{ab(2-q)(4-\alpha -q)}S_{\mu ,\alpha }^{\frac{2^{*}( \alpha )}{2}}}{2^{*}(\alpha )-q} \biggr]^{\frac{2}{6-3\alpha }}\leq \Vert u \Vert \leq \biggl[\frac{ (2^{*}(\alpha )-q )\lambda \vert f\vert _{\infty }C _{R_{0},\beta ,q}}{2(2-\alpha )\sqrt{2ab} S_{\mu ,\beta }^{ \frac{q}{2}}} \biggr]^{\frac{2}{6-\alpha -2q}}. $$

On the other hand, using (2.1) and (2.2) again we have

$$\begin{aligned} a(2-q)\Vert u\Vert ^{2} \leq& a(2-q)\Vert u\Vert ^{2}+b(4-\alpha -q)\Vert u\Vert ^{4-\alpha } \\ =& \bigl(2^{*}(\alpha )-q \bigr) \int_{\mathbb{R}^{3}}\frac{\vert u\vert ^{2^{*}( \alpha )}}{\vert x\vert ^{\alpha }}\,dx \\ \leq& \bigl(2^{*}(\alpha )-q \bigr)S_{\mu , \alpha }^{-\frac{2^{*}(\alpha )}{2}} \Vert u\Vert ^{2^{*}(\alpha )}, \end{aligned}$$

and by (1.5)

$$\begin{aligned} a(4-2\alpha )\Vert u\Vert ^{2} \leq& a(4-2 \alpha )\Vert u\Vert ^{2}+b(2-\alpha )\Vert u\Vert ^{4-\alpha } \\ =& \bigl(2^{*}(\alpha )-q \bigr)\lambda \int_{\mathbb{R}^{3}}\frac{f(x)\vert u\vert ^{q}}{\vert x\vert ^{ \beta }} \\ \leq& \bigl(2^{*}(\alpha )-q \bigr)\lambda \vert f\vert _{\infty }C_{R_{0}, \beta ,q} S_{\mu ,\beta }^{-\frac{q}{2}}\Vert u\Vert ^{q}, \end{aligned}$$

which yields that

2.4 $$ \biggl[\frac{a(2-q)S_{\mu ,\alpha }^{\frac{2^{*}(\alpha )}{2}}}{2^{*}( \alpha )-q} \biggr]^{\frac{1}{4-2\alpha }}\leq \Vert u \Vert \leq \biggl[\frac{ (2^{*}(\alpha )-q )\lambda \vert f\vert _{\infty }C_{R_{0},\beta ,q}}{a(4-2 \alpha ) S_{\mu ,\beta }^{\frac{q}{2}}} \biggr]^{\frac{1}{2-q}}. $$

Combining (2.3) and (2.4), we obtain $\lambda \geq \Lambda_{1}=\max \{\lambda_{1},\lambda_{2}\}$, which is a contradiction. Hence $\mathcal{N}^{0}=\emptyset $ for any $0<\lambda <\Lambda_{1}=\max \{\lambda_{1},\lambda_{2}\}$. The proof is complete. □

To find solutions of (1.1), it is necessary to consider whether $\mathcal{N}^{\pm }$ are nonempty.

### Lemma 2.4

*Assume* (F) *and for any* $0<\lambda <\Lambda_{1}$, *then for any* $u\in D^{1,2}(\mathbb{R}^{3})\backslash \{0\}$ *there exist* $t_{0}>0$ *and unique* $t^{+}$ *and* $t^{-}$ *with* $0< t^{+}< t_{0}< t^{-}$ *such that* $t^{\pm }u\in \mathcal{N}^{\pm }$ *and*

$$ J\bigl(t^{+}u\bigr)=\inf_{0\leq t\leq t_{0}}J(tu) \quad \textit{and} \quad J\bigl(t^{-}u\bigr)= \sup_{t\geq t_{0}}J(tu). $$

### Proof

Compared with the results in [6], the proof is standard after some simple modifications and we omit it. □

From Lemma 2.3, we know that $\mathcal{N}=\mathcal{N}^{+} \cup \mathcal{N}^{-}$ for any $0<\lambda <\Lambda_{1}\triangleq \max \{\lambda_{1},\lambda_{2}\}$. Moreover, by Lemma 2.4 we have $\mathcal{N}^{\pm }\neq \emptyset $ and by Lemma 2.1 we may define

$$ m=\inf_{u\in \mathcal{N}}J(u),\qquad m^{+}=\inf _{u\in \mathcal{N}^{+}}J(u),\qquad m^{-}=\inf_{u\in \mathcal{N}^{-}}J(u). $$

Then we have the following result.

### Lemma 2.5

*Under the assumptions of Theorem *1.2, *we have*

(i) *If* $0<\lambda <\Lambda_{1}=\max \{\lambda_{1}.\lambda_{2}\}$, *then* $m^{+}<0$;
(ii) *If* $0<\lambda <\Lambda_{2}\triangleq \max \{{q\lambda_{1}}/{\sqrt{2(4- \alpha )}},{q\lambda_{2}}/{2}\}$, *then there exists* $d_{0}>0$ *independent of* *b* *such that* $m^{-}>d_{0}$. *In particular*, *we have* $m^{+}=m<0<m^{-}$.

### Proof

(i) For any $u\in \mathcal{N}^{+}$, by (2.1) we know

$$ a(2-q)\Vert u\Vert ^{2}+b(4-\alpha -q)\Vert u\Vert ^{4-\alpha } > \bigl(2^{*}(\alpha )-q \bigr) \int_{\mathbb{R}^{3}}\frac{\vert u\vert ^{2^{*}(\alpha )}}{\vert x\vert ^{\alpha }}\,dx, $$

which implies that

$$\begin{aligned} J(u) & =-\frac{a(2-q)}{2q}\Vert u\Vert ^{2}- \frac{b(4-\alpha -q)}{q(4-\alpha )} \Vert u\Vert ^{4-\alpha } +\frac{2^{*}(\alpha )-q}{2^{*}(\alpha )q} \int_{\mathbb{R}^{3}}\frac{\vert u\vert ^{2^{*}(\alpha )}}{\vert x\vert ^{\alpha }}\,dx \\ &< \frac{a(2-q) (2-2^{*}(\alpha ) )}{2q2^{*}(\alpha )}\Vert u\Vert ^{2}+ \frac{b(4-\alpha -q)(\alpha -2)}{q(4-\alpha )2^{*}(\alpha )}\Vert u \Vert ^{4- \alpha }< 0. \end{aligned}$$

Thus we obtain that $m^{+}<0$.

(ii) To end the proof, we split it into the following two cases.

*Case* 1: $0<\lambda <{q\lambda_{1}}/{\sqrt{2(4-\alpha )}}$.

Similar to (2.3), we can derive

2.5 $$ \Vert u\Vert > \biggl[\frac{2\sqrt{ab(2-q)(4-\alpha -q)}S_{\mu ,\alpha }^{\frac{2^{*}( \alpha )}{2}}}{2^{*}(\alpha )-q} \biggr]^{\frac{2}{6-3\alpha }} \quad \text{for any } u\in \mathcal{N}^{-}. $$

Then, for any $u\in \mathcal{N}^{-}\subset \mathcal{N}$ and by (1.5), we have that

2.6 $$\begin{aligned} J(u) & =\frac{a (2^{*}(\alpha )-2)}{22^{*}(\alpha )}\Vert u\Vert ^{2}+\frac{b(2- \alpha )}{(4-\alpha )2^{*}(\alpha )}\Vert u\Vert ^{4-\alpha } - \frac{\lambda (2^{*}(\alpha )-q)}{q2^{*}(\alpha )} \int_{\mathbb{R}^{3}}\frac{f(x)\vert u\vert ^{q}}{\vert x\vert ^{ \beta }}\,dx \\ &\geq \Vert u\Vert ^{q} \biggl[ \frac{2(2-\alpha )\sqrt{2ab}}{\sqrt{2(4- \alpha )}2^{*}(\alpha )}\Vert u \Vert ^{\frac{6-\alpha -2q}{2}}-\frac{\lambda (2^{*}(\alpha )-q)}{q2^{*}(\alpha )} \vert f\vert _{\infty }C_{R_{0},\beta ,q} S_{\mu ,\beta }^{-\frac{q}{2}} \biggr]. \end{aligned}$$

Combining (2.5) and (2.6), we know that if $\lambda <{q\lambda_{1}}/{\sqrt{2(4-\alpha )}}$, there exists $d_{0}>0$ independent of *b* such that $m^{-}\geq d_{0}$.

*Case* 2: $0<\lambda <{q\lambda_{2}}/{2}$.

Similar to (2.4), we can derive

2.7 $$ \Vert u\Vert > \biggl[\frac{a(2-q)S_{\mu ,\alpha }^{\frac{2^{*}(\alpha )}{2}}}{2^{*}( \alpha )-q} \biggr]^{\frac{1}{4-2\alpha }} \quad \text{for any } u\in \mathcal{N}^{-}. $$

Then, for any $u\in \mathcal{N}^{-}\subset \mathcal{N}$ and by (1.5), we have that

2.8 $$\begin{aligned} J(u) & =\frac{a (2^{*}(\alpha )-2)}{22^{*}(\alpha )}\Vert u\Vert ^{2}+\frac{b(2- \alpha )}{(4-\alpha )2^{*}(\alpha )}\Vert u\Vert ^{4-\alpha } - \frac{\lambda (2^{*}(\alpha )-q)}{q2^{*}(\alpha )} \int_{\mathbb{R}^{3}}\frac{f(x)\vert u\vert ^{q}}{\vert x\vert ^{ \beta }}\,dx \\ &\geq \Vert u\Vert ^{q} \biggl[ \frac{a(4-2\alpha )}{22^{*}(\alpha )}\Vert u \Vert ^{2-q}-\frac{\lambda (2^{*}(\alpha )-q)}{q2^{*}(\alpha )} \vert f\vert _{ \infty }C_{R_{0},\beta ,q} S_{\mu ,\beta }^{-\frac{q}{2}} \biggr]. \end{aligned}$$

Combining (2.7) and (2.8), we know that if $\lambda <{q\lambda_{2}}/{2}$, there exists $d_{0}>0$ independent of *b* such that $m^{-}\geq d_{0}$. The proof is complete. □

## Proof of Theorem 1.2

In this section, we prove Theorem 1.2. Using Ekeland’s variational principle [10] and the argument in [6, Lemma 5.2], we have the following result.

### Lemma 3.1

*Under the assumptions of Theorem *1.2, *we have*

(i) *If* $0<\lambda <\Lambda_{1}$, *then* $J(u)$ *has a* $(PS)_{m}$ *sequence* $\{u_{n}\}\subset \mathcal{N}$;
(ii) *If* $0<\lambda <\Lambda_{2}$, *then* $J(u)$ *has a* $(PS)_{m^{-}}$ *sequence* $\{u_{n}\}\subset \mathcal{N}^{-}$.

The following lemma provides the interval where the $(PS)$ condition holds for $J(u)$.

### Lemma 3.2

*If* $\lambda \in (0,\Lambda_{*})$, *any* $(PS)_{c}$ *sequence of* $J(u)$ *contains a strongly convergent subsequence whenever* $c< c_{ \mu ,\alpha }^{*}-C_{0}\lambda^{{2}/{(2-q)}}$, *where*

3.1 $$\begin{aligned} c^{*}_{\mu ,\alpha } =& \frac{a(2-\alpha )}{2(3-\alpha )}S_{\mu ,\alpha } \biggl(\frac{bS_{\mu ,\alpha }^{\frac{4-\alpha }{2}}+\sqrt{b^{2}S _{\mu ,\alpha }^{4-\alpha }+4aS_{\mu ,\alpha }}}{2} \biggr)^{\frac{2}{2- \alpha }} \\ &{}+\frac{b(2-\alpha )}{2(3-\alpha )(4-\alpha )}S_{\mu ,\alpha }^{\frac{4- \alpha }{2}} \biggl( \frac{bS_{\mu ,\alpha }^{\frac{4-\alpha }{2}}+\sqrt{b ^{2}S_{\mu ,\alpha }^{4-\alpha }+4aS_{\mu ,\alpha }}}{2} \biggr) ^{\frac{4- \alpha }{2-\alpha }} \end{aligned}$$

*and* $C_{0}$ *is a positive constant given by Lemma *3.3 *below*.

### Proof

Let $\{u_{n}\}\subset D^{1,2}(\mathbb{R}^{3})$ be a $(PS)_{c}$ sequence of $J(u)$, and we conclude that $\{u_{n}\}$ is bounded in $D^{1,2}( \mathbb{R}^{3})$. In fact

$$\begin{aligned} c+1+o(1)\Vert u_{n}\Vert &\geq J(u_{n})- \frac{1}{4-\alpha }\bigl\langle J^{\prime }(u_{n}),u_{n} \bigr\rangle \\ &\geq \frac{a(2-\alpha )}{2(4-\alpha )}\Vert u_{n}\Vert ^{2}- \frac{4-\alpha -q}{q(4-\alpha )}\lambda \vert f\vert _{\infty }C_{R_{0},\beta ,q} S_{\mu , \beta }^{-\frac{q}{2}}\Vert u_{n}\Vert ^{q}, \end{aligned}$$

which yields that $\{u_{n}\}$ is bounded in $D^{1,2}(\mathbb{R}^{N})$ since $1< q<2$. Up to a subsequence if necessary, there exists $u\in D^{1,2}(\mathbb{R}^{3})$ such that $u_{n}\rightharpoonup u$ in $D^{1,2}(\mathbb{R}^{3})$, $u_{n}\to u$ in $L^{r}_{\text{loc}}( \mathbb{R}^{3})$ for $r\in [1,2^{*}(\alpha ))$ and $u_{n}\to u$ a.e. in $\mathbb{R}^{3}$. Next we prove that $u_{n}\to u$ in $D^{1,2}( \mathbb{R}^{3})$.

By the concentration compactness principle [22], there exist a countable set Γ, a set of different points $\{x_{j}\} \subset \mathbb{R}^{3}\backslash \{0\}$, nonnegative real numbers $\mu_{x_{j}}$, $\nu_{x_{j}}$ for $j\in \Gamma $, and nonnegative real numbers $\mu_{0}$, $\gamma_{0}$, and $\nu_{0}$ such that

$$\begin{aligned}& \vert \nabla u_{n}\vert ^{2} \rightharpoonup d\widetilde{\mu }\geq \vert \nabla u\vert ^{2}+ \sum _{j\in \Gamma }\mu_{x_{j}}\delta_{x_{j}}+{ \mu_{0}}\delta_{0}, \\& {u_{n}^{2}} {\vert x\vert ^{-2}} \rightharpoonup d\gamma ={u^{2}} {\vert x\vert ^{-2}}+{ \gamma_{0}} \delta_{0}, \\& {\vert u_{n}\vert ^{2^{*}(\alpha )}} {\vert x\vert ^{-\alpha }}\rightharpoonup d\nu ={\vert u\vert ^{2^{*}( \alpha )}} {\vert x\vert ^{-\alpha }}+\sum_{j\in \Gamma } \nu_{x_{j}}\delta_{x _{j}}+{\nu_{0}} \delta_{0}, \end{aligned}$$

where $\delta_{x}$ is the Dirac mass at $x\in \mathbb{R}^{3}$. Without loss of generality, we only consider the possibility of concentration at the singular point $0\in \mathbb{R}^{3}$. To do it, for any $\epsilon >0$, we let $x_{j}\notin B_{\epsilon }(0)$ for all $j\in \Gamma $ and choose $\varphi^{\epsilon }$ to be a smooth cut-off function such that $0\leq \varphi^{\epsilon }\leq 1$, $\varphi^{ \epsilon }\equiv 0$ when $x\in B_{\epsilon }^{c}(0)$, $\varphi^{ \epsilon }\equiv 1$ when $x\in B_{{\epsilon }/{2}}(0)$ and $|\nabla \varphi^{\epsilon }|\leq {4}/{\epsilon }$. Then

3.2 $$ \begin{aligned} &\lim_{\epsilon \to 0} \lim_{n\to \infty } \int_{\mathbb{R}^{3}}\vert \nabla u_{n}\vert ^{2}\varphi^{\epsilon }\,dx =\lim_{\epsilon \to 0} \int_{\mathbb{R}^{3}}\varphi^{\epsilon }\,d\widetilde{\mu }\geq \mu_{0}, \\ &\lim_{\epsilon \to 0}\lim_{n\to \infty } \int_{\mathbb{R}^{3}}{u_{n} ^{2}} {\vert x\vert ^{-2}}\varphi^{\epsilon }\,dx =\lim_{\epsilon \to 0} \int_{\mathbb{R}^{3}}\varphi^{\epsilon }\,d\gamma = \gamma_{0}, \\ &\lim_{\epsilon \to 0}\lim_{n\to \infty } \int_{\mathbb{R}^{3}}{\vert u_{n}\vert ^{2^{*}( \alpha )}} { \vert x\vert ^{-\alpha }}\varphi^{\epsilon }\,dx =\lim _{\epsilon \to 0} \int_{\mathbb{R}^{3}}\varphi^{\epsilon }\,d\nu =\nu_{0}, \\ &\lim_{\epsilon \to 0}\lim_{n\to \infty } \int_{\mathbb{R}^{3}}u_{n} \nabla u_{n} \varphi^{\epsilon }\,dx=0, \\ &\lim_{\epsilon \to 0}\lim_{n\to \infty } \int_{\mathbb{R}^{3}}{f(x)\vert u _{n}\vert ^{q}} {\vert x\vert ^{-\beta }}\varphi^{\epsilon }\,dx=0. \end{aligned} $$

Since $\{u_{n}\}$ is bounded, using (3.2) we have

$$\begin{aligned} 0 =& \lim_{\epsilon \to 0}\lim_{n\to \infty }\bigl\langle J^{\prime }(u _{n}),u_{n}\varphi^{\epsilon }\bigr\rangle \\ =& \lim_{\epsilon \to 0}\lim_{n\to \infty } \biggl\{ \bigl(a+b \Vert u_{n}\Vert ^{2- \alpha }\bigr) \biggl( \int_{\mathbb{R}^{3}}\vert \nabla u_{n}\vert ^{2}\varphi^{\epsilon }+ u_{n}\nabla u_{n} \varphi^{\epsilon }-\mu \frac{u_{n}^{2}}{\vert x\vert ^{2}}\varphi^{\epsilon }\,dx \biggr) \\ &{} - \int_{\mathbb{R}^{3}}\frac{\vert u_{n}\vert ^{2^{*}(\alpha )}}{\vert x\vert ^{\alpha }} \varphi_{j}^{\epsilon } \,dx -\lambda \int_{\mathbb{R}^{3}}\frac{f(x)\vert u_{n}\vert ^{q}}{\vert x\vert ^{\beta }}\varphi^{\epsilon }\,dx \biggr\} \\ \geq & a(\mu_{0}-\mu \gamma_{0})+b(\mu_{0}-\mu \gamma_{0})^{{(4- \alpha )}/{2}}-\nu_{0}. \end{aligned}$$

In view of Sobolev inequality (1.3), that is, $S_{\mu , \alpha }^{ {2^{*}(\alpha )}/{2}}\nu_{0}\leq (\mu_{0}-\mu \gamma_{0})^{ {2^{*}(\alpha )}/{2}}$, we derive

$$ S_{\mu ,\alpha }^{-(3-\alpha )}(\mu_{0}-\mu \gamma_{0})^{2-\alpha }-b( \mu_{0}-\mu \gamma_{0})^{\frac{2-\alpha }{2}}-a\geq 0, $$

which gives that

$$ (\mu_{0}-\mu \gamma_{0})\geq S_{\mu ,\alpha } \biggl( \frac{bS_{\mu , \alpha }^{\frac{4-\alpha }{2}} +\sqrt{b^{2}S_{\mu ,\alpha }^{{4- \alpha }}+4aS_{\mu ,\alpha }}}{2} \biggr)^{\frac{1}{2-\alpha }}. $$

Therefore we have

$$\begin{aligned} & c+o(1) \\ &\quad =J(u_{n})-\frac{1}{4-\alpha }\bigl\langle J^{\prime }(u_{n}),u_{n} \bigr\rangle \\ &\quad =\frac{a(2-\alpha )}{2(4-\alpha )}\Vert u_{n}\Vert ^{2}+\frac{2-\alpha }{2(4- \alpha )(3-\alpha )} \int_{\mathbb{R}^{3}}\frac{\vert u_{n}\vert ^{2^{*}(\alpha )}}{\vert x\vert ^{\alpha }}\,dx-\frac{4-\alpha -q}{q(4-\alpha )} \lambda \int_{\mathbb{R}^{3}}\frac{f(x)\vert u_{n}\vert ^{q}}{\vert x\vert ^{\beta }}\,dx \\ &\quad \geq \frac{a(2-\alpha )}{2(4-\alpha )}\bigl(\mu_{0}-\mu \gamma_{0}+\Vert u\Vert ^{2}\bigr)+\frac{2-\alpha }{2(4-\alpha )(3-\alpha )} \nu_{0} -\frac{4- \alpha -q}{q(4-\alpha )} \lambda \vert f\vert _{\infty }C_{R_{0},\beta ,q} S _{\mu ,\beta }^{-\frac{q}{2}}\Vert u \Vert ^{q} \\ &\quad \geq \frac{a(2-\alpha )}{2(4-\alpha )}(\mu_{0}-\mu \gamma_{0}) +\frac{2- \alpha }{2(4-\alpha )(3-\alpha )} \bigl[a(\mu_{0}-\mu \gamma_{0})+b(\mu _{0}-\mu \gamma_{0})^{\frac{4-\alpha }{2}} \bigr]-C_{0} \lambda^{\frac{2}{2-q}} \\ &\quad =\frac{a(2-\alpha )}{2(3-\alpha )}(\mu_{0}-\mu \gamma_{0})+ \frac{b(2- \alpha )}{2(4-\alpha )(3-\alpha )}(\mu_{0}-\mu \gamma_{0})^{\frac{4- \alpha }{2}}-C_{0} \lambda^{\frac{2}{2-q}} \geq c_{\mu ,\alpha }^{*}-C _{0} \lambda^{\frac{2}{2-q}}, \end{aligned}$$

a contradiction! Hence we have

$$ \int_{\mathbb{R}^{3}}\vert u_{n}\vert ^{2^{*}(\alpha )} \vert x\vert ^{-\alpha }\,dx\to \int_{\mathbb{R}^{3}}\vert u\vert ^{2^{*}(\alpha )}\vert x\vert ^{-\alpha }\,dx, $$

which together with (1.5) implies

$$ \int_{\mathbb{R}^{3}}f(x)\vert u_{n}\vert ^{q} \vert x\vert ^{-\beta }\,dx\to \int_{\mathbb{R}^{3}}f(x)\vert u\vert ^{q}\vert x\vert ^{\beta }\,dx. $$

Hence there holds

$$\begin{aligned} o(1) & =\bigl\langle J^{\prime }(u_{n})-J^{\prime }(u),u_{n}-u \bigr\rangle \\ & =\bigl(a+b\Vert u_{n}\Vert ^{2-\alpha }\bigr) (u_{n},u_{n}-u)-\bigl(a+b\Vert u\Vert ^{2-\alpha } \bigr) (u,u _{n}-u)+o(1) \\ &=\bigl(a+b\Vert u_{n}\Vert ^{2-\alpha }\bigr) (u_{n}-u,u_{n}-u)+b\bigl(\Vert u_{n}\Vert ^{2-\alpha }-\Vert u\Vert ^{2-\alpha }\bigr) (u,u_{n}-u)+o(1) \\ &\geq a\Vert u_{n}-u\Vert ^{2}+o(1), \end{aligned}$$

which yields that $u_{n}\to u$ in $D^{1,2}(\mathbb{R}^{3})$. The proof is complete. □

To apply in Lemma 3.2, we have the following result.

### Lemma 3.3

*Under the assumptions of Theorem *1.2, *there holds*

$$ \sup_{t\geq 0}J(tU)< c_{\mu ,\alpha }^{*}-C_{0} \lambda^{\frac{2}{2-q}},\qquad C_{0}=\frac{a(2-\alpha )(2-q)}{2q(4-\alpha )} \biggl[ \frac{(4- \alpha -q)\vert f\vert _{\infty }C_{R_{0},\beta ,q} }{a(2-\alpha )S_{\mu , \beta }^{ {q}/{2}}} \biggr]^{\frac{2}{2-q}}>0 $$

*for any* $\lambda \in (0,\Lambda_{*})$. *In particular*, $m^{-}< c_{ \mu ,\alpha }^{*}-C_{0}\lambda^{\frac{2}{2-q}}$ *for any* $\lambda \in (0,\Lambda_{*})$.

### Proof

For $c_{\mu ,\alpha }>0$ given by (3.1), we have that

$$ c_{\mu ,\alpha }^{*}-C_{0}\lambda^{\frac{2}{2-q}}>0 \quad \text{for any } \lambda \in (0,\lambda_{3}). $$

Let us define

$$\begin{aligned} g(t) =&\frac{a}{2}t^{2}\Vert U\Vert ^{2}+ \frac{b}{4-\alpha }t^{4-\alpha }\Vert U\Vert ^{4-\alpha } - \frac{t^{2^{*}(\alpha )}}{2^{*}(\alpha )} \int_{\mathbb{R}^{3}}\frac{\vert U\vert ^{2^{*}(\alpha )}}{\vert x\vert ^{\alpha }}\,dx \\ \triangleq& C_{1}t^{2}+C_{2}t^{4-\alpha }-C_{3}t^{2^{*}(\alpha )}, \quad t\geq 0. \end{aligned}$$

As a consequence of (1.4), we have

$$ C_{1}=\frac{a}{2}S^{\frac{3-\alpha }{2-\alpha }}_{\mu ,\alpha }, \qquad C _{2}=\frac{b}{4-\alpha }S^{ \frac{(4-\alpha )(3-\alpha )}{2(2-\alpha )}}_{\mu ,\alpha },\qquad C _{3}=\frac{1}{2^{*}(\alpha )}S^{\frac{3-\alpha }{2-\alpha }}_{\mu , \alpha }. $$

By some elementary calculations, we have

$$ g^{\prime }(t)=2C_{1}t+C_{2}(4-\alpha )t^{3-\alpha }-C_{3}2^{*}( \alpha )t^{5-2\alpha }=0, \qquad t\geq 0, $$

which is equivalent to

$$ 2C_{1}+C_{2}(4-\alpha )t^{2-\alpha }-C_{3}2^{*}( \alpha )t^{4-2\alpha }=0, \qquad t\geq 0. $$

Since $4-2\alpha =2(2-\alpha )$, we know that $g^{\prime }(t)=0$ has a unique root, that is,

$$ \widetilde{t}=S_{\mu ,\alpha }^{-\frac{1}{2(2-\alpha )}} \biggl(\frac{bS _{\mu ,\alpha }^{\frac{4-\alpha }{2}} +\sqrt{b^{2}S_{\mu ,\alpha } ^{{4-\alpha }}+4aS_{\mu ,\alpha }}}{2} \biggr)^{\frac{1}{2-\alpha }}>0. $$

Therefore we can conclude that

$$\begin{aligned} \max_{t\geq 0}g(t) &=g(\widetilde{t})=C_{1} \widetilde{t}^{2}+C_{2} \widetilde{t}^{4-\alpha }- \frac{2C_{1} \widetilde{t}^{2}+C_{2}(4- \alpha \widetilde{t}^{4-\alpha }}{2^{*}(\alpha )} \\ & = \frac{2-\alpha }{3-\alpha }C_{1}\widetilde{t}^{2} + \frac{2- \alpha }{2(3-\alpha )}C_{2}\widetilde{t}^{4-\alpha }=c^{*}_{\mu , \alpha }, \end{aligned}$$

which implies that

3.3 $$ J(tU)=g(t)-\frac{t^{q}}{q}\lambda \int_{\mathbb{R}^{3}}\frac{f(x)\vert U\vert ^{q}}{\vert x\vert ^{ \beta }}\,dx \leq c^{*}_{\mu ,\alpha }- \frac{t^{q}}{q}\lambda \int_{\mathbb{R}^{3}}\frac{f(x)\vert U\vert ^{q}}{\vert x\vert ^{\beta }}\,dx \quad \text{for any } t\geq 0. $$

Since $J(0)=0$, there exists $t_{1}\in (0,1)$ only depending on $\lambda_{3}$ such that

$$ \max_{0\leq t\leq t_{1}}J(tU)< c_{\mu ,\alpha }^{*}-C_{0} \lambda^{\frac{2}{2-q}} \quad \text{for any } \lambda \in (0,\lambda _{3}). $$

On the other hand, by (3.3) we have that

$$ \max_{t\geq t_{1}}J(tU)\leq c_{\mu ,\alpha }^{*}- \frac{t_{1}^{q}}{q} \lambda \int_{\mathbb{R}^{3}}f(x)\vert U\vert ^{q}\vert x\vert ^{-\beta }\,dx, $$

which gives

$$ \max_{t\geq t_{1}}J(tU)< c_{\mu ,\alpha }^{*}-C_{0} \lambda^{ \frac{2}{2-q}} \quad \text{for any } 0< \lambda < \lambda_{4}. $$

Finally, we can deduce that

$$ \max_{t\geq 0}J(tU)< c_{\mu ,\alpha }^{*}-C_{0} \lambda^{\frac{2}{2-q}} \quad \text{for any } 0< \lambda < \Lambda_{*}. $$

Since $U\in D^{1,2}(\mathbb{R}^{3})\backslash \{0\}$, by Lemma 2.4 there exists unique $t_{U}^{\pm }$ such that $t_{U}^{ \pm }U\in \mathcal{N}^{\pm }$. Consequently, we have $m^{-}\leq J(t _{U}^{-} U)\leq \max_{t\geq 0}J(tU)$, which completes the proof. □

Now, we establish the existence of a local minimum for $J(u)$ on $\mathcal{N}$.

### Proposition 3.4

*Assume* (F), $\mu \in [0,1/4)$, $\alpha ,\beta \in [0,2)$, *and* $q\in (1,2)$, *then for any* $\lambda \in (0,\Lambda_{*})$ *there exists* $u_{\lambda }\in D^{1,2}(\mathbb{R}^{3})$ *such that*

(i) $u_{\lambda }$ *is a positive solution of* (1.1) *and* $J(u_{\lambda })=m=m^{+}$;
(ii) $\|u_{\lambda }\|\to 0$ *as* $\lambda \to 0^{+}$.

### Proof

(i) In view of Proposition 3.1(i), any minimizing sequence $\{u_{n}\}\subset \mathcal{N}$ of *m* can be chosen as a $(PS)_{m}$ sequence of $J(u)$, that is,

$$ J(u_{n})\to m+o(1) \quad \text{and}\quad J^{\prime }(u_{n})=o(1) \quad \text{as } n\to \infty . $$

By Lemma 2.1, we know that $\{u_{n}\}$ is bounded in $D^{1,2}(\mathbb{R}^{3})$. Going to a subsequence if necessary, there exists $u_{\lambda }\in D^{1,2}(\mathbb{R}^{3})$ such that $u_{n} \rightharpoonup u_{\lambda }$ in $D^{1,2}(\mathbb{R}^{3})$. It follows from the definitions of *m* and $m^{\pm }$ that $m\leq m^{\pm }$. Hence $u_{n}\to u_{\lambda }$ in $D^{1,2}(\mathbb{R}^{3})$ by Lemmas 3.2–3.3, then $J(u_{\lambda })=m$ and $J^{\prime }(u_{\lambda })=0$. Since $m\leq m^{+}<0$, we can derive $u_{\lambda }$ is a nontrivial solution of (1.1) by Lemma 2.2. By the fact that $J(u)$ is translation invariant, we know that $J(|u_{\lambda }|)=J(u_{\lambda })=m$ and $J^{\prime }(|u_{\lambda }|)=J^{\prime }(u_{\lambda })=0$. By using Harnack’s inequality [26], it follows that $u_{\lambda }(x)>0$ in $\mathbb{R}^{3}$ and then $u_{\lambda }$ is a positive solution of (1.1). We now claim that $u_{\lambda } \in \mathcal{N}^{+}$. Indeed, we argue it indirectly and assume $u_{\lambda }\in \mathcal{N}^{-}$ by Lemma 2.3. It follows from Lemma 2.4 that there exist unique $t_{\lambda }^{+}$ and $t_{\lambda }^{-}$ such that $t_{\lambda }^{\pm }u_{\lambda }\in \mathcal{N}^{\pm }$ with $0< t_{\lambda }^{+}< t_{\lambda }^{-}\equiv 1$. By the same idea used in [6, Lemma 4.2], we know that $\varphi_{u_{\lambda }}(t)=J(tu_{\lambda })$ is strictly increasing on $(t_{\lambda }^{+},t_{\lambda }^{-})$ and hence

$$ m^{+}\leq J\bigl(t_{\lambda }^{+} u_{\lambda } \bigr)< J\bigl(t_{\lambda }^{-} u_{ \lambda } \bigr)=J(u_{\lambda })=m\leq m^{+}, $$

a contradiction! So, we can obtain $u_{\lambda }\in \mathcal{N}^{+}$, which implies that $m^{+}\leq J(u_{\lambda })=m\leq m^{+}$. Consequently, the proof of (i) is complete.

(ii) Since $u_{\lambda }\in \mathcal{N}^{+}$, then similar to (2.3) and (2.4) we have

$$ \Vert u_{\lambda }\Vert < \min \biggl\{ \biggl[\frac{ (2^{*}(\alpha )-q )\lambda \vert f\vert _{\infty }C_{R_{0},\beta ,q}}{2(2-\alpha ) \sqrt{2ab} S_{\mu ,\beta }^{\frac{q}{2}}} \biggr]^{ \frac{2}{6-\alpha -2q}}, \biggl[\frac{ (2^{*}(\alpha )-q )\lambda \vert f\vert _{\infty }C_{R_{0},\beta ,q}}{a(4-2\alpha ) S_{\mu ,\beta }^{ \frac{q}{2}}} \biggr]^{\frac{1}{2-q}} \biggr\} , $$

which yields $\|u_{\lambda }\|\to 0$ as $\lambda \to 0^{+}$. The proof is complete. □

Next, we establish the existence of a local minimum for $J(u)$ on $\mathcal{N}^{-}$.

### Proposition 3.5

*Assume* (F), $\mu \in [0,1/4)$, $\alpha ,\beta \in [0,2)$, *and* $q\in (1,2)$, *then for any* $\lambda \in (0,\Lambda_{**})$ *there exists* $U_{\lambda }\in D^{1,2}(\mathbb{R}^{3})$ *such that*

(i) $J(U_{\lambda })=m^{-}$;
(ii) $U_{\lambda }$ *is a positive solution of* (1.1).

### Proof

It follows from Proposition 3.1(ii) that there exists a $(PS)_{m^{-}}$ sequence of $J(u)$,

$$ J(u_{n})\to m^{-}+o(1) \quad \text{and}\quad J^{\prime }(u_{n})=o(1) \quad \text{as } n\to \infty . $$

Hence $\{u_{n}\}$ is bounded in $D^{1,2}(\mathbb{R}^{3})$ by Lemma 2.1 and there exists $U_{\lambda }\in D^{1,2}(\mathbb{R} ^{3})$ such that $u_{n}\rightharpoonup U_{\lambda }$ in $D^{1,2}( \mathbb{R}^{3})$ in the sense of a subsequence. Using Lemmas 3.2–3.3, we obtain $u_{n}\to U_{ \lambda }$ in $D^{1,2}(\mathbb{R}^{3})$ and then $J(U_{\lambda })=m ^{-}$ and $J^{\prime }(U_{\lambda })=0$. In view of Lemma 2.2 and Lemma 2.5(ii), we know that $U_{\lambda }$ is a nontrivial solution of (1.1). Similar to Proposition 3.4, we have that $U_{\lambda }$ is positive. The proof is complete. □

We are now in a position to complete the proof of Theorem 1.2.

### Proof of Theorem 1.2

The part (i) is a corollary of Proposition 3.4. If $\lambda \in (0,\Lambda_{**})$, we can obtain two positive solutions $u_{\lambda }\in \mathcal{N}^{+}$ and $U_{\lambda }\in \mathcal{N} ^{-}$ of (1.1) by Propositions 3.4–3.5. The definitions of $\mathcal{N}^{\pm }$ give us $\mathcal{N}^{+}\cap \mathcal{N}^{-}=\emptyset $, then we know that $u_{\lambda }$ and $U_{\lambda }$ are two different positive solutions of (1.1). □

## Asymptotic behavior as $b\searrow 0^{+}$

In this section, we regard $b\in (0,1]$ as a parameter in problem (1.1) and analyze the convergence property. To do it, we have to prove that problem (1.1) admits at least two nontrivial solutions again. We introduce the following variational functional:

$$ J_{b}(u)=\frac{a}{2}\Vert u\Vert ^{2}+ \frac{b}{4-\alpha }\Vert u\Vert ^{4-\alpha } -\frac{1}{2^{*}( \alpha )} \int_{\mathbb{R}^{3}}\vert u\vert ^{2^{*}(\alpha )}\vert x\vert ^{-\alpha }\,dx-\frac{ \lambda }{q} \int_{\mathbb{R}^{3}}f(x)\vert u\vert ^{q}\vert x\vert ^{-\beta }\,dx $$

to emphasize the independence of $b\in (0,1]$.

Now we will verify that the functional $J_{b}(u)$ exhibits the mountain pass geometry.

### Lemma 4.1

*The functional* $J_{b}(u)$ *satisfies the mountain pass geometry around* $0\in D^{1,2}(\mathbb{R}^{3})$ *for any* $\lambda \in (0, \lambda_{5})$, *that is*,

(i) *there exist* $\delta ,\rho >0$ *independent of* *b* *such that* $J_{b}(u) \geq \delta >0$ *when* $\|u\|=\rho $;
(ii) *there exists* $e\in D^{1,2}(\mathbb{R}^{3})$ *with* $\|e\|>\rho $ *such that* $J(e)<0$.

### Proof

(i) It follows from (1.3) and (1.5) that

$$\begin{aligned} J_{b}(u) &\geq \Vert u\Vert ^{q} \biggl( \frac{a}{2}\Vert u\Vert ^{2-q}-\frac{1}{2^{*}( \alpha )}S_{\mu ,\alpha }^{-\frac{2^{*}(\alpha )}{2}} \Vert u\Vert ^{2^{*}( \alpha )-q} -\frac{\lambda }{q}\vert f\vert _{\infty }C_{R_{0},\beta ,q} S_{ \mu ,\beta }^{-\frac{q}{2}} \biggr) \\ &\geq \rho^{q} \biggl\{ \frac{a(2^{*}(\alpha )-2)}{2(2^{*}(\alpha )-q)}\rho^{2-q} - \frac{ \lambda }{q}\vert f\vert _{\infty }C_{R_{0},\beta ,q} S_{\mu ,\beta }^{- \frac{q}{2}} \biggr\} , \end{aligned}$$

where

$$ \rho = \biggl[\frac{a2^{*}(\alpha )S_{\mu ,\alpha }^{\frac{2^{*}(\alpha )}{2}}(2-q)}{2(2^{*}(\alpha )-q)} \biggr]^{\frac{1}{2^{*}(\alpha )-2}}. $$

Therefore there exists $\delta >0$ such that $J_{b}(u)\geq \delta >0$ when $\|u\|=\rho >0$ for any $\lambda \in (0,\lambda_{5})$.

(ii) Choosing $u_{0}\in D^{1,2}(\mathbb{R}^{3})\backslash \{0\}$, then since $4-\alpha <2^{*}(\alpha )$ one has

$$ J_{b}(tu_{0})\leq \frac{a}{2}t^{2} \Vert u_{0}\Vert ^{2}+\frac{b}{4-\alpha }t ^{4-\alpha } \Vert u_{0}\Vert ^{4-\alpha } -\frac{t^{2^{*}(\alpha )}}{2^{*}( \alpha )} \int_{\mathbb{R}^{3}}\frac{\vert u_{0}\vert ^{2^{*}(\alpha )}}{\vert x\vert ^{ \alpha }}\,dx\to -\infty \quad \text{as } t \to +\infty . $$

Hence letting $e=t_{0}u_{0}\in D^{1,2}(\mathbb{R}^{3})\backslash \{0 \}$ with $t_{0}$ sufficiently large, we have $\|e\|>\rho $ and $J(e)<0$. The proof is complete. □

By Lemma 4.1 and the mountain pass theorem in [28], a $(PS)$ sequence of the functional $J(u)$ at the level

4.1 $$ c_{b}:=\inf_{\gamma \in \Gamma }\max _{t\in [0,1]}J_{b}\bigl(\gamma (t)\bigr) \geq \delta >0 $$

can be constructed, where the set of paths is defined as

$$ \Gamma_{b}:= \bigl\{ \gamma \in C\bigl([0,1],D^{1,2}\bigl( \mathbb{R}^{3}\bigr)\bigr):\gamma (0)=0, J_{b}\bigl(\gamma (1)\bigr)< 0 \bigr\} . $$

In other words, there exists a sequence $\{u_{n}\}\subset D^{1,2}( \mathbb{R}^{3})$ such that

4.2 $$ J_{b}(u_{n})\to c_{b},\qquad J_{b}^{\prime }(u_{n})\to 0\quad \text{as } n\to \infty . $$

### Remark 4.2

By (4.1), we can conclude that $c_{b}< c_{\mu ,\alpha }-C_{0}\lambda^{2/(2-q)}$ for any $\lambda \in (0,\lambda_{M})$. In fact, in view of the proof of Lemma 3.3, we obtain

$$ \sup_{t\geq 0}J(tU)< c_{\mu ,\alpha }^{*}-C_{0} \lambda^{\frac{2}{2-q}} $$

for any $\lambda \in (0,\lambda_{M})$. As the proof of Lemma 4.1(ii), there exists sufficiently large $t_{U}>0$ such that $J_{b}(t_{U}U)<0$. Hence let $\gamma_{0}(t)=tt_{U}U\in \Gamma _{b}$, then $c_{b}\leq \sup_{t\geq 0}J(tU)$, which yields $c_{b}< c _{\mu ,\alpha }-C_{0}\lambda^{2/(2-q)}$ for any $\lambda \in (0, \lambda_{M})$.

To obtain a solution with negative energy, we introduce the following lemma.

### Lemma 4.3

(Ekeland’s variational principle [10], Theorem 1.1)

*Let* *V* *be a complete metric space and* $F:V\to \mathbb{R}\cup \{+\infty \}$ *be lower semicontinuous*, *bounded from below*. *Then*, *for any* $\epsilon >0$, *there exists some point* $v\in V$ *with*

$$ F(v)\leq \inf_{V}F+\epsilon , \qquad F(w)\geq F(v)-\epsilon d(v,w)\quad \textit{for all } w\in V. $$

Now, we establish the existence of multiple solutions of (1.1).

### Proposition 4.4

*Assume* (F), $\mu \in [0,1/4)$, $\alpha ,\beta \in [0,2)$, *and* $q\in (1,2)$, *then equation* (1.1) *has at least two positive solutions* $u_{b}^{1}$ *and* $u_{b}^{2}$ *satisfying*

$$ J_{b}\bigl(u_{b}^{2}\bigr)< 0< J_{b} \bigl(u_{b}^{1}\bigr),\quad \forall \lambda \in (0, \lambda_{M}). $$

### Proof

Let $\{u_{n}\}\subset D^{1,2}(\mathbb{R}^{3})$ satisfy (4.2), by Lemma 3.2 and Remark 4.2 we derive there exists $u_{b}^{1}\in D^{1,2}(\mathbb{R}^{3})$ such that $J_{b}^{\prime }(u_{b}^{1})=0$ and $J_{b}(u_{b}^{1})=c_{b}>0$.

On the other hand, for $\rho >0$ given by Lemma 4.1(i), we define

$$ \overline{B}_{\rho }=\bigl\{ u\in D^{1,2}\bigl( \mathbb{R}^{3}\bigr),\Vert u\Vert \leq \rho \bigr\} ,\qquad \partial B_{\rho }=\bigl\{ u\in D^{1,2}\bigl(\mathbb{R}^{3} \bigr),\Vert u\Vert = \rho \bigr\} , $$

clearly $\overline{B}_{\rho }$ is a complete metric space with the distance $d(u,v)=\|u-v\|$. It is obvious that the functional $J_{b}$ is lower semicontinuous and bounded from below on $\overline{B}_{\rho }$. We claim that

4.3 $$ \widetilde{c}_{b} :=\inf \bigl\{ J_{b}(u):u \in \overline{B}_{\rho }\bigr\} < 0. $$

Indeed, choosing a nonnegative function $\psi \in C_{0}^{\infty }( \mathbb{R}^{3})$, we have

$$ \lim_{t\to 0}\frac{J_{b}(t\psi )}{t^{q}}=-\frac{\lambda }{q} \int_{\mathbb{R}^{3}}\frac{f(x)\vert \psi \vert ^{q}}{\vert x\vert ^{\beta }}\,dx< 0. $$

Therefore there exists a sufficiently small $t_{\psi }>0$ such that $\|t_{\psi }\psi \|\leq \rho $ and $J_{b}(t_{\psi }\psi )<0$, which imply that (4.3) holds. By Lemma 4.3, for any $n\in N$, there exists $\widetilde{u}_{n}$ such that

$$ \widetilde{ c}_{b}\leq J_{b}(\widetilde{u}_{n}) \leq \widetilde{c}_{b} +\frac{1}{n}, \quad \text{and} \quad J_{b}(v)\geq J_{b}(\widetilde{u}_{n})- \frac{1}{n}\Vert \widetilde{u}_{n}-v\Vert , \quad \forall v \in \overline{B}_{ \rho }. $$

Then a standard procedure gives that $\{u_{n}\}$ is a bounded $(PS)_{\widetilde{ c}_{b}}$ sequence of $J_{b}$. Therefore, by Lemma 3.2 and (4.3), there exists $u_{b}^{2}\in D^{1,2}(\mathbb{R}^{3})$ such that $J_{b}^{\prime }(u _{b}^{2})=0$ and $J_{b}(u_{b}^{2})=\widetilde{ c}_{b}<0$. It is similar to Proposition 3.4 that $u_{b}^{1}$ and $u_{b}^{2}$ are positive. □

For $b\in (0,1]$, we can obtain two sequences $\{u_{b}^{1}\}$ and $\{u_{b}^{2}\}$ of solutions of (1.1) by Proposition 4.4, that is,

4.4 $$ J^{\prime }_{b}\bigl(u_{b}^{1} \bigr)=0,\qquad J_{b}\bigl(u_{b}^{1} \bigr)=c_{b}, $$

and

4.5 $$ J^{\prime }_{b}\bigl(u_{b}^{2} \bigr)=0,\qquad J_{b}\bigl(u_{b}^{2}\bigr)= \widetilde{c_{b}}, $$

The variational functional corresponding to (1.6) is given by

$$ J_{0}(u)=\frac{a}{2}\Vert u\Vert ^{2}- \frac{1}{2^{*}(\alpha )} \int_{\mathbb{R}^{3}}\frac{\vert u\vert ^{2^{*}(\alpha )}}{\vert x\vert ^{\alpha }}\,dx -\frac{ \lambda }{q} \int_{\mathbb{R}^{3}}\frac{f(x)\vert u\vert ^{q}}{\vert x\vert ^{\beta }}\,dx $$

which is of class of $C^{1}$ due to [28]. For any $b\in (0,1]$, we have

$$\begin{aligned} c^{*}_{\mu ,\alpha } \leq& \frac{a(2-\alpha )}{2(3-\alpha )}S_{\mu , \alpha } \biggl(\frac{S_{\mu ,\alpha }^{\frac{4-\alpha }{2}}+\sqrt{S _{\mu ,\alpha }^{4-\alpha }+4aS_{\mu ,\alpha }}}{2} \biggr)^{\frac{2}{2- \alpha }} \\ &{}+\frac{(2-\alpha )}{2(3-\alpha )(4-\alpha )}S_{\mu ,\alpha }^{\frac{4- \alpha }{2}} \biggl( \frac{S_{\mu ,\alpha }^{\frac{4-\alpha }{2}}+\sqrt{S _{\mu ,\alpha }^{4-\alpha }+4aS_{\mu ,\alpha }}}{2} \biggr) ^{\frac{4- \alpha }{2-\alpha }}\triangleq M_{0}< +\infty , \end{aligned}$$

where $M_{0}$ is independent of *b*.

### Proof of Theorem 1.4

To end the proof clearly, we will split it into several steps.

*Step* 1: There exist four constants independent of $b\in (0,1]$ such that

4.6 $$ 0< \delta \leq c_{b}< M_{0}-C_{0} \lambda^{\frac{2}{2-q}} \quad \text{and}\quad -C_{0} \lambda^{\frac{2}{2-q}}\leq \widetilde{c_{b}}\leq -\frac{\lambda }{2q} \int_{\mathbb{R}^{3}}\frac{f(x)\vert \psi_{0}\vert ^{q}}{\vert x\vert ^{\beta }}\,dx< 0. $$

In fact, the constant $\delta >0$ given by Lemma 4.1 is independent of any $b>0$, then by (4.1) we have that $J_{b}(u_{b}^{1})\geq \delta $. On the other hand, using (1.5) we have

$$\begin{aligned} J_{b}\bigl(u_{b}^{2}\bigr) &=J_{b} \bigl(u_{b}^{2}\bigr)-\frac{1}{4-\alpha }\bigl\langle J^{ \prime }_{b}\bigl(u_{b}^{2} \bigr),u_{b}^{2}\bigr\rangle \\ &\geq \frac{a(2-\alpha )}{2(4-\alpha )}\bigl\Vert u_{b}^{2}\bigr\Vert ^{2}-\frac{2^{*}( \alpha )-q}{q2^{*}(\alpha )} \lambda \vert f\vert _{\infty }C_{R_{0},\beta ,q} S _{\mu ,\beta }^{-\frac{q}{2}}\bigl\Vert u_{b}^{2} \bigr\Vert ^{q}\geq -C_{0} \lambda^{\frac{p}{p-q}}. \end{aligned}$$

Let $\psi_{0}\in C_{0}^{\infty }(\mathbb{R}^{3})$ satisfy $\|\psi_{0} \|\leq (2qC_{0}/|f|_{\infty }C_{R_{0},\beta ,q})^{\frac{1}{q}} \lambda^{\frac{1}{2-q}}S_{\mu ,\beta }^{\frac{1}{2}}$ and since

$$ \lim_{t\to 0}\frac{J_{b}(t\psi_{0})}{t^{q}}=-\frac{\lambda }{q} \int_{\mathbb{R}^{3}}\frac{f(x)\vert \psi_{0}\vert ^{q}}{\vert x\vert ^{\beta }}\,dx< 0, $$

we can let $t_{0}>0$ such that $\|t_{0}\psi \|\leq \rho $, where $\rho >0$ is given by Lemma 4.1(ii). Therefore we can obtain

$$ \widetilde{c_{b}}=\inf \bigl\{ J_{b}(u):u\in \overline{B}_{\rho }\bigr\} \leq -\frac{ \lambda }{2q} \int_{\mathbb{R}^{3}}\frac{f(x)\vert \psi_{0}\vert ^{q}}{\vert x\vert \beta }\,dx< 0. $$

So the proof of Step 1 is complete.

*Step* 2: The sequences $\{u_{b}^{i}\}$ ($i\in \{1,2\}$) contain strongly convergent subsequences.

By (4.4) and (4.5), we know that $\{u_{b}^{i} \}$ ($i\in \{1,2\}$) are $(PS)$ sequences of the functionals $J_{b}(u)$. We claim that $\{u_{b}^{i}\}$ ($i\in \{1,2\}$) are bounded. In fact,

$$\begin{aligned} M &> J_{b}\bigl(u_{b}^{i} \bigr)=J_{b}\bigl(u_{b}^{i}\bigr)- \frac{1}{4-\alpha }\bigl\langle J _{b}^{\prime } \bigl(u_{b}^{i}\bigr),u_{b}^{i}\bigr\rangle \\ &\geq \frac{a(2-\alpha )}{2(4-\alpha )}\bigl\Vert u_{b}^{i}\bigr\Vert ^{2}-\frac{2^{*}( \alpha )-q}{q2^{*}(\alpha )} \lambda \vert f\vert _{\infty }C_{R_{0},\beta ,q} S _{\mu ,\beta }^{-\frac{q}{2}}\bigl\Vert u_{b}^{i}\bigr\Vert ^{q}, \end{aligned}$$

which yields that $\{u_{b}^{i}\}$ are bounded in $D^{1,2}(\mathbb{R} ^{3})$ since $1< q<2$. With (4.4) and (4.5) in hand, we can see Lemma 3.2 as a special case to show that the sequences $\{u_{b}^{i}\}$ ($i\in \{1,2\}$) contain strongly convergent subsequences with $c_{b}<\frac{1}{2}(2-\alpha )(3-\alpha )^{-1}(aS _{\mu ,\alpha })^{(3-\alpha )/(2-\alpha )}$. Hence there exist subsequences still denoted by themselves and $u^{i}\in D^{1,2}( \mathbb{R}^{3})$ such that $u_{b}^{i}\to u^{i}$ in $D^{1,2}( \mathbb{R}^{3})$ as $b\to 0^{+}$ for $i\in \{1,2\}$. Therefore, $\forall \varphi \in C_{0}^{\infty }(\mathbb{R}^{3})$ we have

$$\begin{aligned} 0 =&\bigl(a+b\bigl\Vert u_{b}^{i} \bigr\Vert ^{2-\alpha }\bigr) \int_{\mathbb{R}^{3}}\nabla u_{b}^{i} \nabla \varphi -\mu {u_{b}^{i}\varphi } {\vert x\vert ^{-2}}\,dx- \int_{\mathbb{R} ^{3}}{\bigl\vert u_{b}^{i}\bigr\vert ^{2^{*}(\alpha )-2}u_{b}^{i}\varphi } {\vert x\vert ^{-\alpha }}\,dx \\ &{}-\lambda \int_{\mathbb{R}^{3}}\frac{f(x)\vert u_{b}^{i}\vert ^{q-2}u_{b}^{i} \varphi }{\vert x\vert ^{\beta }}\,dx \\ \to& a \int_{\mathbb{R}^{3}}\nabla u^{i}\nabla \varphi -\mu {u^{i} \varphi } {\vert x\vert ^{-2}}\,dx- \int_{\mathbb{R}^{3}}{\bigl\vert u^{i}\bigr\vert ^{2^{*}(\alpha )-2}u ^{i}\varphi } {\vert x\vert ^{-\alpha }}\,dx \\ &-\lambda \int_{\mathbb{R}^{3}}{f(x)\bigl\vert u^{i}\bigr\vert ^{q-2}u^{i}\varphi } {\vert x\vert ^{-\beta }}\,dx \end{aligned}$$

as $b\to 0^{+}$, which yields that $u^{i}\in D^{1,2}(\mathbb{R}^{3})$ are solutions of (1.6) for $i\in \{1,2\}$.

*Step* 3: $J_{0}(u^{2})<0<J_{0}(u^{1})$.

Indeed,

$$ J_{0}\bigl(u^{1}\bigr)=\lim_{b\to 0^{+}}J_{b} \bigl(u_{b}^{1}\bigr)\geq \delta >0 $$

and

$$ J_{0}\bigl(u^{2}\bigr)=\lim_{b\to 0^{+}}J_{b} \bigl(u_{b}^{2}\bigr)\leq - \frac{\lambda }{2q} \int_{\mathbb{R}^{3}}\frac{f(x)\vert \psi_{0}\vert ^{q}}{\vert x\vert ^{ \beta }}\,dx< 0. $$

Summing the above three steps, we obtain that $u^{1}$ and $u^{2}$ are two nontrivial solutions of (1.6). The proof is complete. □

## Conclusion

This paper is concerned with the qualitative analysis of solutions of a nonlocal problem with Sobolev–Hardy exponent of Kirchhoff type. Meanwhile, it seems that the study of Kirchhoff type equation involving Hardy term and singular nonlinearity via the Nehari manifold and fibering maps is new.
